# A toolkit for recombinant production of seven human EGF family growth factors in active conformation

**DOI:** 10.1038/s41598-022-09060-9

**Published:** 2022-03-23

**Authors:** Arthur Schveitzer Ferreira, Amanda Lopacinski, Michel Batista, Priscila Mazzocchi Hiraiwa, Beatriz Gomes Guimarães, Nilson Ivo Tonin Zanchin

**Affiliations:** 1 Laboratory of Structural Biology and Protein Engineering, Carlos Chagas Institute, FIOCRUZ Paraná, Curitiba, PR Brazil; 2 Cellular and Molecular Biology Graduate Program, Federal University of Paraná, Curitiba, PR Brazil; 3 Mass Spectrometry Facility RPT02H, Carlos Chagas Institute, FIOCRUZ Paraná, Curitiba, PR Brazil

**Keywords:** Biotechnology, Expression systems

## Abstract

Epidermal growth factors (EGF) play a wide range of roles in embryogenesis, skin development, immune response homeostasis. They are involved in several pathologies as well, including several cancer types, psoriasis, chronic pain and chronic kidney disease. All members share the structural EGF domain, which is responsible for receptor interaction, thereby initiating transduction of signals. EGF growth factors have intense use in fundamental research and high potential for biotechnological applications. However, due to their structural organization with three disulfide bonds, recombinant production of these factors in prokaryotic systems is not straightforward. A significant fraction usually forms inclusion bodies. For the fraction remaining soluble, misfolding and incomplete disulfide bond formation may affect the amount of active factor in solution, which can compromise experimental conclusions and biotechnological applications. In this work, we describe a reliable procedure to produce seven human growth factors of the EGF family in *Escherichia coli*. Biophysical and stability analyses using limited proteolysis, light scattering, circular dichroism and nanoDSF show that the recombinant factors present folded and stable conformation. Cell proliferation and scratch healing assays confirmed that the recombinant factors are highly active at concentrations as low as 5 ng/ml.

## Introduction

Several diseases are associated to disruption of growth factor homeostasis. This is so for the family of epidermal growth factors (EGF) whose altered expression has been linked to several pathogeneses, such as lung adenocarcinoma and several other cancer types, chronic pain, chronic kidney disease and psoriasis^1–5^. Signaling by EGF growth factors is mediated by the ErbB family of G protein-coupled receptors, which comprises four members (ErbB1 to ErbB4). ErbBs have three conserved domains: an ectodomain responsible for ligand binding, a transmembrane domain, and a cytoplasmic tyrosine kinase domain responsible for signal transduction. Binding of the EGF growth factors to the receptors is mediated by the EGF domain, leading to homo or heteroligomerization of the receptors and activating cross-phosphorylation of their intracellular C-terminal domain. Receptor activation may trigger signal transduction to several pathways involved in differentiation, proliferation, migration or survival, with a wide range of targets including PLCϒ, STAT5, SHIP1, Abl, PTP-2c, STAT1, Syk, Vav2, RasA1, SH3BGRL, Grb2, Src, Crk, Nck, Shc and Cb1^6^. ErbB receptors are found in many cell types and tissues, such as blood cells, hepatocytes, cardiac cells, as well as in epithelial, endothelial, nervous, bone and adipose tissues^7^. EGF growth factors are likewise present in many cell types and tissues, such as epithelial cells, fibroblasts, blood cells, platelets, endothelial cells, colon, glands, mineralized tissues, among others^8^.

Structurally, all EGF growth factors share the EGF domain, which comprises six cysteine residues, with a conserved nucleus of constant number of residues, CX7CX4-5CX10-13CXCX8C. The six conserved cysteines form three disulfide bonds with the C1-C3, C2-C4, and C5-C6 combination. This arrangement results in formation of the A, B, and C loops, which divide the structure in the N-terminal portion, composed of loops A and B and the C-terminal portion, comprising loop C. Loops B and C are separated by a single residue, the so-called hinge residue, which allows flexibility between the two segments^9^. Both the N- and C-regions have distinct roles and are important for receptor selectivity due to differences in affinity of each region for the many different receptors. It is, thus, important that the entire domain is well-structured for efficient binding^10,11^.

EGF growth factors can bind to different ErbB receptors and induce formation of different receptor dimers. However, they are usually grouped according to their biological activity and receptor specificity^9,12,13^. In this work, we focused on the seven members of the EGF family of growth factors that bind exclusively to ErbB1, also known as HER1 (human EGFR related) and as EGFR (epidermal growth factor receptor)^8,9,12^. In addition to epidermal growth factor (EGF) itself, this group includes amphiregulin (AREG), betacellulin (BTC), epiregulin (EPR), epigen (EPGN), Heparin-binding epidermal growth factor (HBEGF) and the transforming growth factor alpha (TGFα). In human cells, these seven EGF growth factors are synthesized as type I transmembrane precursor proteins, composed of an N-terminal extension, the EGF domain, a membrane juxtaposed portion, a transmembrane domain, and a cytoplasmic C-terminal tail. The EGF precursor protein, however, is an exception, presenting nine repeats of the EGF domain^14^ although, after processing only the one closest to the membrane can actively bind to the receptors. AREG and HBEGF have also a Heparin-Binding domain adjacent to the EGF domain^8^.

ErbB1 ligand EGF growth factors have wide utilization from fundamental research to state-of-the-art therapies^15–17^. However, recombinant production of EGF domain growth factors has been a challenging task, demanding either eukaryotic systems, such as yeast^17,18^, mammalian cell lineages^19–21^, or fusion with ancillary proteins^22–26^ or even in vitro renaturing procedures^27,28^. Although the pro-forms of EGF family members have post-translational modifications even before membrane processing, none of the post-translational modifications is located at the EGF domain, allowing for their production in *Escherichia coli*, a system for which highly tested vectors for regulated expression are available, being also less time-consuming and more cost-effective^29^. Despite these advantages, recombinant production of complex proteins in *E. coli* may lead to incorrect folding, inclusion body formation, disulfide bond mismatch, among other limitations.

Disulfide bond formation is a critical step to obtain functional EGF growth factors. Therefore, many strategies have been attempted to overcome this problem, such as utilization of the *E. coli* Shuffle^30^ and Origami 2 (DE3)^31^ strains to try to control redox environment, secretion to the periplasm^32^, and fusion with chaperone-proteins, such as oleosin^33^, glutathione S-transferase^34^, SUMO^33^, B1 domain of streptococcal protein G^34^, protein disulfide isomerase (PDI)^35^, N-utilization substance protein A^35^, maltose-binding protein^35^, b'a' domain of PDI (PDIb'a')^35^, ELK16^36^ and thioredoxin (Trx)^37^. In general, these studies have demonstrated the requirement of a fusion partner for proper folding of the EGF growth factors, which can be efficiently separated by proteolytic cleavage. Among the various fusion proteins used in previous studies, Trx and SUMO were shown to be efficient fusion partners for *E. coli* expression of hEPR^22,37^ and hHBEGF^25^, respectively.

In addition to the technical limitations related to choosing the appropriate fusion partner for *E. coli* expression, there is also a need for consistent information on the procedures to purify the EGF domain growth factors, as well as for a description of the biophysical methods ensuring the correct structural conformation and, functional demonstration of their activity. Therefore, in this work, we developed a simple and easily reproducible method for recombinant expression, purification, biophysical characterization, and demonstration of activity for the seven members of the EGF growth factor family that are known to bind ErbB1. The same experimental set up and reagents can be used for production of all seven EGF factors. We demonstrate by using biophysical analyses that the recombinant factors present folded and stable conformation. Furthermore, we demonstrate their usefulness in cell proliferation and scratch healing assays.

## Results

### Expression and purification of the EGF growth factors

**Figure 1:**
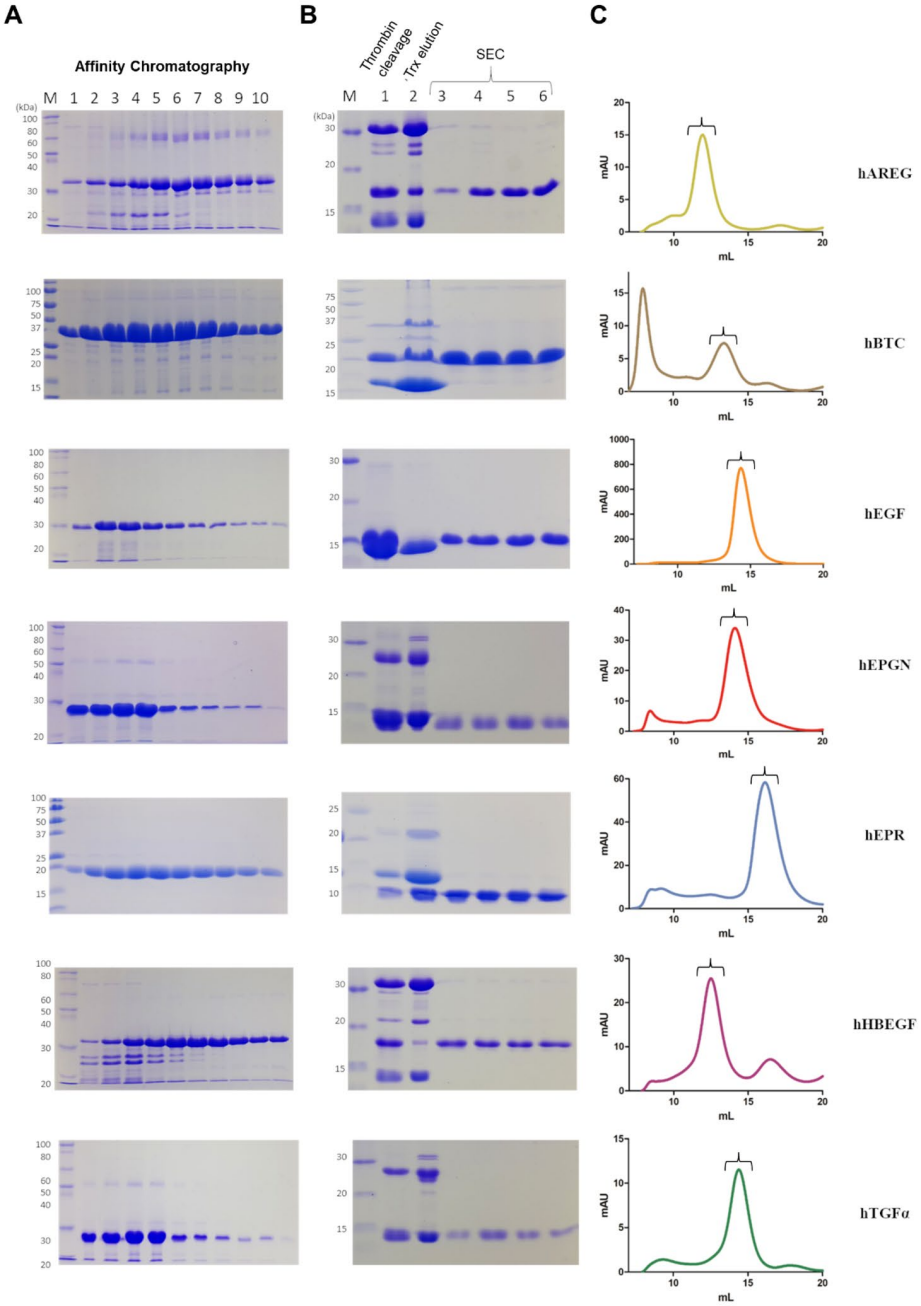
Purification of the EGF growth factors. (**A**) Images of Coomassie stained SDS–polyacrylamide gels showing the peak fractions of the initial affinity chromatography (1 to 10). (**B**) Images of Coomassie stained SDS–polyacrylamide gels showing the result of thrombin protease digestion (lane 1), elution fraction from the purification of Trx-his6 after thrombin cleavage (lane 2) and peak fractions after the size exclusion chromatography (SEC) (lanes 3–6). Sizes (kDa) of the molecular mass markers (M) are indicated on the left. Only the relevant parts of the gels are shown. Original uncropped images of the gels are presented in the Supplementary Information file, Figure S1. (**C**) Elution profiles of the size exclusion chromatography analyses on a Superdex 75 10/300 GL column. Brackets indicate the region of the peak analyzed by SDS-PAGE in (B). Each row represents the purification of the EGF growth factor indicated on the right.

Expression of the EGF domain growth factors hAREG, hBTC, hEGF, hEPGN, hEPR, hHBEGF and hTGFα in fusion with Trx in the *E. coli* strain Shuffle performed overnight at 16 °C was highly efficient. An initial immobilized nickel affinity chromatography was used to purify the fusion proteins (Fig. 1A). Following separation of the histidine-tagged Trx from the EGF growth factors by thrombin digestion, the histidine-tagged Trx was captured on an immobilized nickel column. The growth factors recovered in the flow through were further fractionated by size-exclusion chromatography (Fig. 1B,C). hEGF, hEPGN, hEPR and hTGFα eluted in a single peak with the expected volume for a monomeric conformation while hAREG and hHBEGF eluted with lower volumes indicating dimerization as previously described^38^. hBTC, on the other hand, showed partial aggregation with approximately 50% of the sample eluting in the void volume. Only the hBTC fractions eluting in the expected volume for monomeric conformation were used in the assays. The secondary peak in the hHBEGF profile corresponds to contaminants. All factors showed a high yield and purity > 95% after the last step of chromatography (Fig. 1B). 500-mL cultures yielded approximately 20 mg of Trx-growth factor fusion proteins after the first affinity purification chromatography. The final yield after digestion of 10 mg of the fusion proteins with thrombin and size exclusion chromatography was: 1–3 mg for hAREG, 3–5 mg for hBTC, 6–8 mg for hEGF, 3–5 mg for hEPGN, 3–5 mg for hEPR, 1–3 mg for hHBEGF and 1–3 mg for hTGFα.

### Stability and conformational analysis of the recombinant EGF growth factors

**Figure 2:**
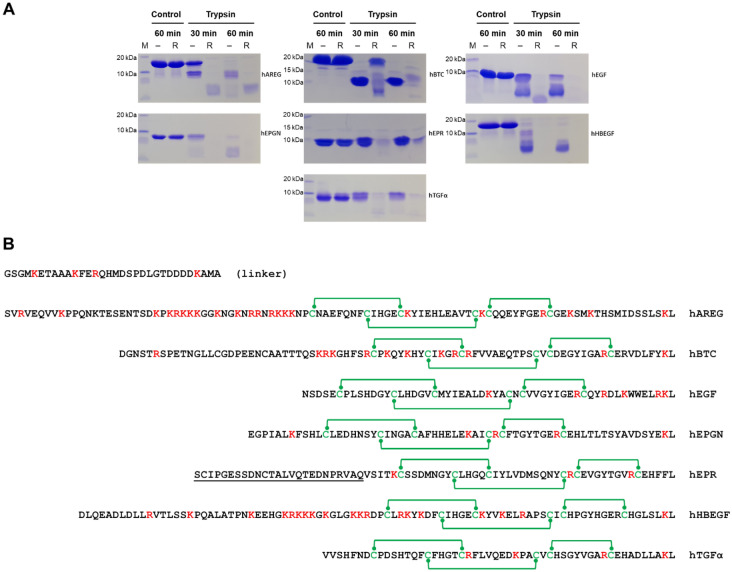
Stability analysis by limited proteolysis using trypsin. The proteolytic assays were performed with samples both untreated and treated with the reducing agent DTT and incubated for 30 and 60 min. Control non-reduced and reduced samples without trypsin treatment were incubated under the same conditions. (**A**) The panels show Coomassie stained SDS–polyacrylamide gels used for the analysis of the proteolysis products and control reactions of the EGF growth factors as identified on the right side of each panel. M, molecular mass markers. -, non-reducing condition. R, reducing condition. Only the relevant parts of the gels are shown. Original uncropped images of the gels are presented in the Supplementary Information file, Figure S2. (**B**) Sequences of the EGF domains with the basic residues target of trypsin marked in red and the cysteine residues marked in green. Green lines indicate the disulfide bonds. The sequence identified by (linker) represents the part of the linker encoded by the vector which remains attached to the growth factors after thrombin digestion. The underlined EPR region indicates the segment of the hEPR propeptide required for stable expression of this EGF growth factor.

The conformation of the seven recombinant EGF growth factors was initially assessed by limited proteolysis assays. Considering that the EGF domain comprises the constant sequence CX7CX4-5CX10-13CXCX8C with disulfide bonds linking the cysteine residues in the combinations C1-C3, C2-C4, C5-C6, the limited proteolysis assays were performed with recombinant factors previously treated with DTT in parallel with control samples that were not treated with reducing agent (Fig. 2A). All reduced samples showed high sensitivity to trypsin, being totally degraded after 30 min of incubation time, except for BTC that showed some resistance, although it also was completely digested after 60 min. On the other hand, in the absence of reducing agents, the growth factors showed more resistance to trypsin proteolysis (Fig. 2A). hEGF, hEPR, and hTGFα were the most resistant, followed by hAREG and hEPGN with hBTC and hHBEGF being more sensitive. Considering only the non-reduced samples, higher resistance to trypsin seems to correlate with the lower number of trypsin cleavage sites found in the EGF domain (Fig. 2B). Except for hEPR, all non-reduced samples showed intermediate bands (Fig. 2A) that we interpreted as the EGF domain nucleus maintained by the disulfide bonds. The intermediate band of hBTC shows particularly high resistance to degradation. In the case of hBTC, this intermediate band remains stable up to 60 min of trypsin treatment. It is unclear why proteolysis of hEPR does not produce such an intermediate band. In conclusion, the higher resistance to proteolysis shown by the growth factors in the absence of treatment with reducing agents gives an indication that the disulfide bonds are formed and possibly also display the right combination.

**Figure 3:**
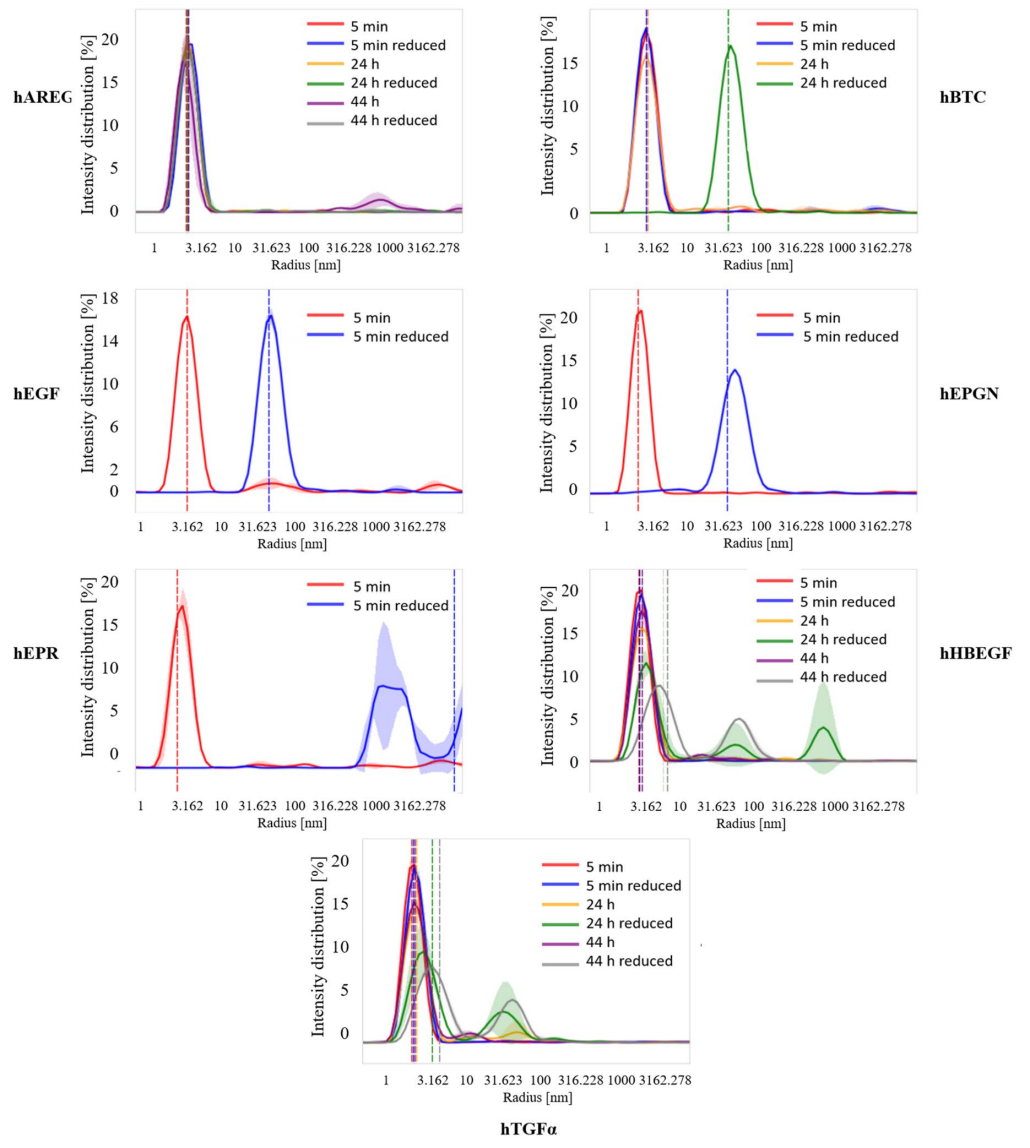
Radius size and aggregation analysis by DLS. Profiles of radius size distribution of the purified EGF growth factors under reducing and non-reducing conditions as determined by dynamic light scattering analyses. The dashed vertical lines represent the center of the cumulative radius of each sample. All assays were performed at 20 °C. Times and treatment are indicated in the figure. The graphics show the intensity distribution (%) as a function of particle radius (nm).

The effect of the reducing agent on the conformation of the growth factors was also investigated by dynamic light scattering (DLS, Fig. 3). Under non-reducing conditions all growth factors showed a single peak with radius size centered at ~ 3 nm. According to the DLS measurements, hEGF, hEPGN and hEPR respond quickly to treatment with the reducing agent. Immediately after adding DTT, the radius size of hEGF and hEPGN shifts from ~ 3 to 30–35 nm while the hEPR radius size increases to ~ 1000 nm, indicating aggregation after reduction of the disulfide bonds (Fig. 3). In the case of hBTC, an effect on radius size, similar to the one seen for hEGF and hEPGN, was detected only after 24 h of incubation with the reducing agent (Fig. 3). Surprisingly, the hAREG radius size was not affected by the reducing agent even after 44 h of incubation while hHBEGF and hTGFα shown partial increase in the radius size after 24 and 44 h of incubation with the reducing agents (Fig. 3). In general, the effect of the reducing agent on the radius size indicates that before reducing the disulfide bonds, the growth factors are properly folded.

**Figure 4:**
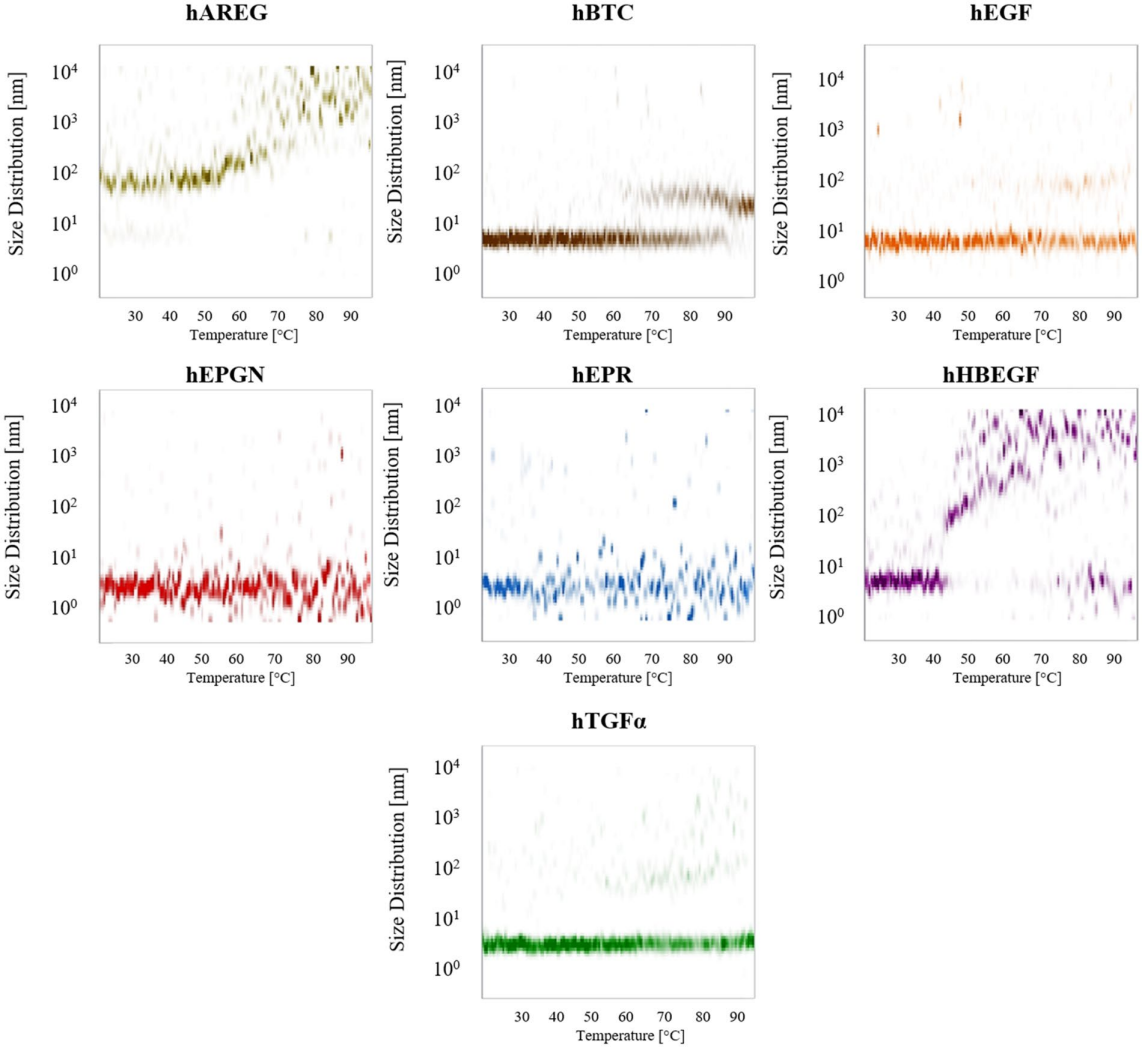
Particle size distributions (nm) for the seven growth factors as a relation of temperature increase from 20 to 95 °C as determined by DLS using non-reduced proteins.

Thermal stability of the growth factors under non-reducing conditions was initially accessed by nano-differential scanning fluorimetry (nanoDSF) combined with parallel radius size analysis by DLS. Since EGF has two tryptophan residues and other EGFs have tyrosine and phenylalanine residues, the fluorescence signal was followed at 330 nm. The fluorescence signals did not show a clear transition between unfolded and folded states with temperature increase (Supplementary Figure S3). For hEGF, HEPGN, hEPR and hTGFα, the radius size did not change significantly indicating that there is no or little aggregation despite temperature increase (Fig. 4). For hAREG and hBTC there is an increase in the particles size at ~ 55 to 60 °C while for hHBEGF starts showing aggregation at ~ 45 °C (Fig. 4). Complete data can be found in the Supplementary Information file.

**Figure 5:**
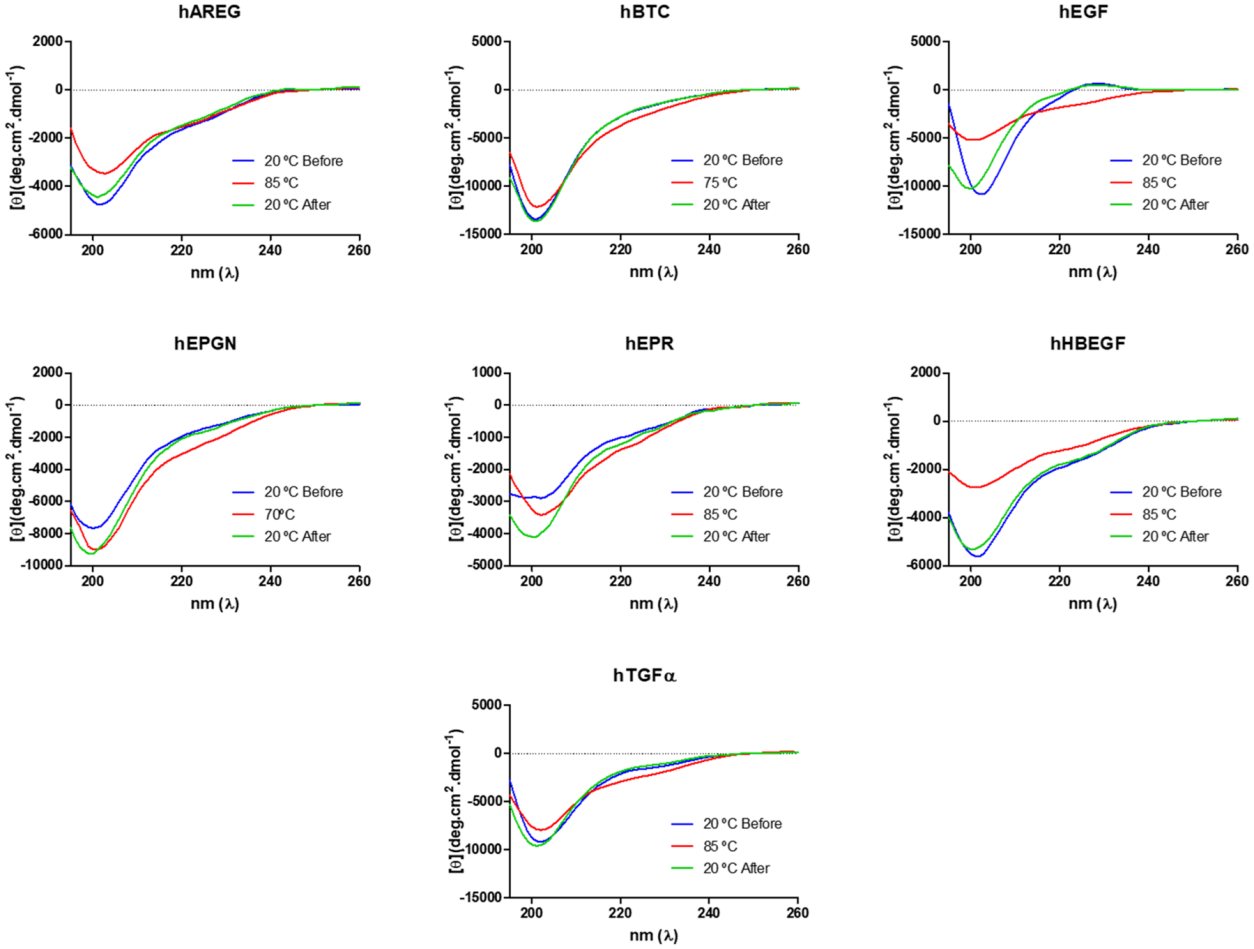
Circular dichroism spectra of the seven EGF growth factors. The blue lines correspond to the spectra acquired at 20 °C, the red lines to the spectra acquired after heating to the indicated temperatures and the green lines correspond to the spectra acquired after the temperature was reduced to 20 °C. The far-UV circular dichroism spectra (195–260 nm) were recorded using a J-815 spectropolarimeter with five accumulations and using a 1 mm path length cell.

The circular dichroism (CD) spectrum for each EGF growth factor under non-reducing conditions was acquired at 20 °C, followed by temperature increase (70–85 °C) and after returning the temperature down to 20 °C (Fig. 5). Similar to the analysis by nanoDSF, temperature increase monitored by CD did not reveal transition states for the EGF growth factors. Actually, the CD spectra of hBTC, hEPGN and hTGFα did not really change with temperature. hAREG, hEGF and hHBEGF showed a reduction of CD signal at 85 °C as compared to the signal at 20 °C. After reducing the temperature, the negative peak of the hEGF and hEPR spectra shifted slightly to a lower wavelength, indicating that they do not return completely to the same conformation observed at 20 °C. The fact that the temperature increase did not produce transition between folded an unfolded states is consistent with the rigid structure of the EGF domains maintained by the three disulfide bonds. The resistance to thermal denaturation also indicates that the disulfide bonds should be formed in the proper combination.

### Functional analysis of the recombinant EGF growth factors

**Figure 6:**
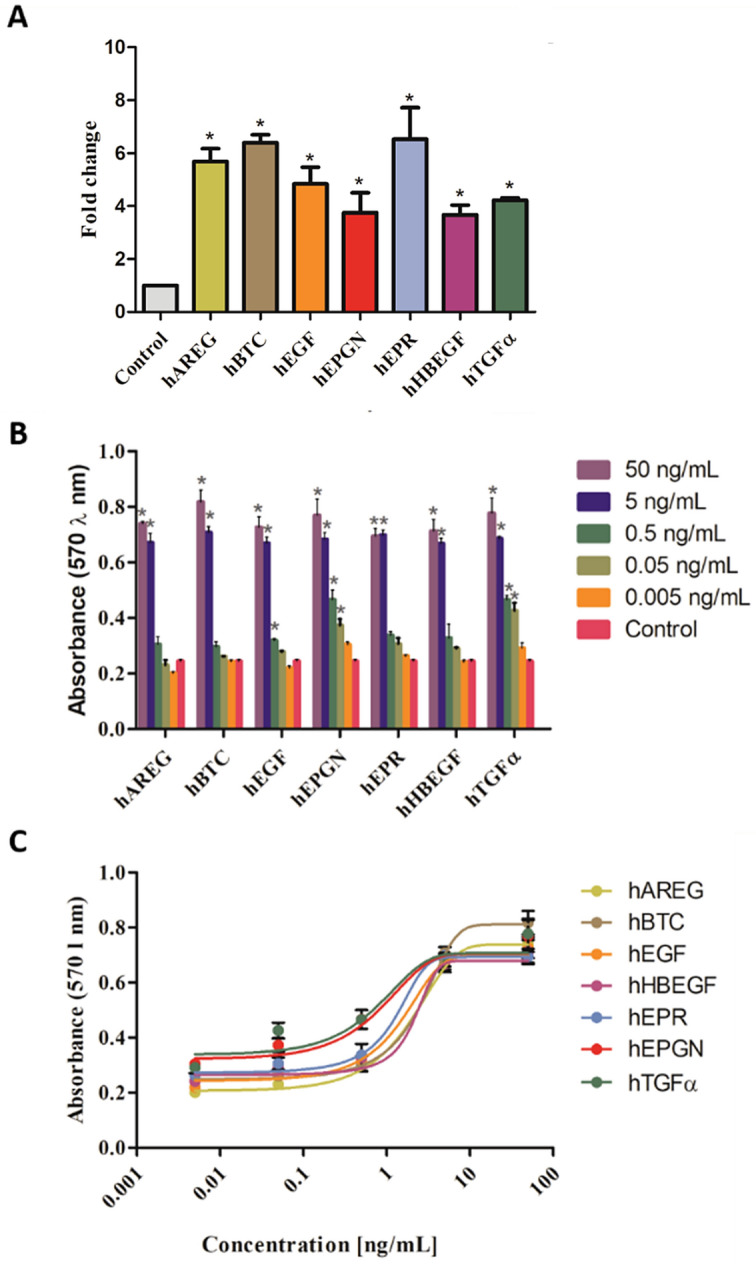
Activity of the seven EGF growth factors on cell proliferation. (**A**) Induction of HeLa cell proliferation by the seven EGF growth factors. Triplicates of HeLa cell cultures were treated with 50 ng/mL of each growth factor and the fold change of the proliferation rate relative to untreated cells (control) was determined after 48 h. The cells were quantified by flow cytometry and normalized using the CountBright™ Absolute Counting Beads method*.* *p < 0.05. (**B**) HeLa cell proliferation induced by the seven EGF growth factors as determined by the MTT assay. Triplicate cultures of HeLa cells were treated with the indicated amounts of the respective EGF growth factors for 48 h and quantified using the MTT assay. *p < 0.05. **(C)** Cell cultures absorbance as a function of the growth factor concentration. The mean values were derived from the assay described in (B) and curves were fitted with nonlinear regression.

The activity of the recombinant EGF growth factors was initially evaluated by cell proliferation assays. Their capacity to stimulate cell proliferation was tested in vitro using HeLa cells with a fixed concentration of 50 ng/mL (Fig. 6A). All factors led to strong induction of HeLa cells proliferation with at least a threefold increase relative to the control untreated cells. hEPR showed the highest induction capacity, followed by hBTC, hAREG, hEGF, hTGFα, hEPGN and, hHBEGF with the lowest stimulation.

A dose response curve was performed using serial dilutions of the recombinant growth factors, starting at 50 ng/mL, and the proliferation of HeLa cells determined indirectly by the MTT method (Fig. 6B,C). The dose–response curve showed that all EGF growth factors induce cell proliferation at the concentrations of 50 and 5 ng/mL. hEPGN, hEPR and hTGFα showed statistically difference from control also at 0.5 ng/mL whereas hEPGN and hTGFα induced cell proliferation at concentration as low as 0.05 ng/mL. These results show that the recombinant EGF growth factors are highly potent to induce cell proliferation.

**Figure 7:**
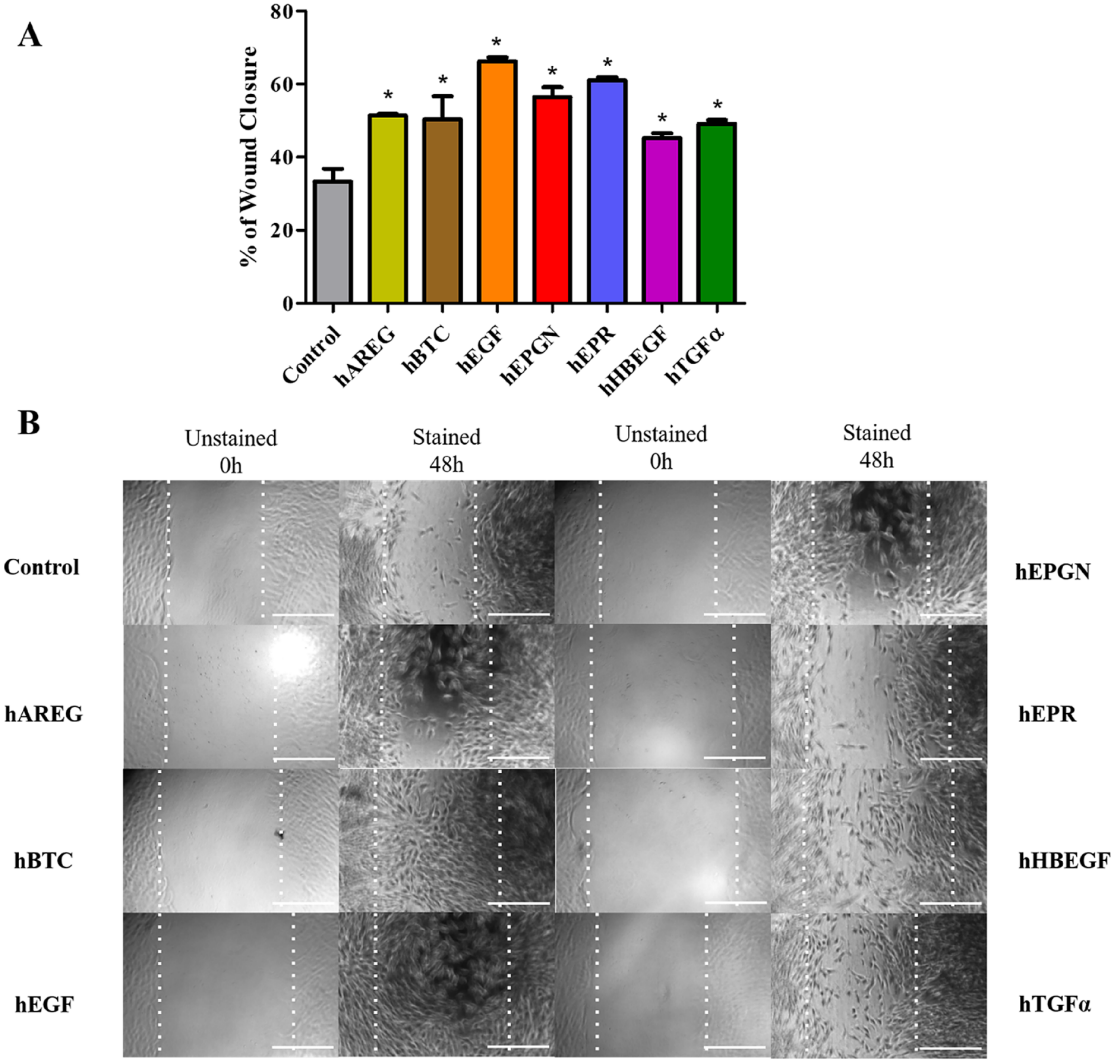
Scratch wound closure induced by the seven EGF growth factors. (**A**) Confluent cultures of NDFH cells were scratched with a sterile pipette tip and treated with 50 ng/mL of each EGF growth factor for 48 h. For quantification of the gap closure rates, the area of the gap at 48 h was compared to the area of the initial gap. Gap areas were determined with the ImageJ software using microscopic images as described in the Methods section. *p < 0.05. (**B**) Representative microscopy images at 100 X magnification acquired at time 0 (unstained living cells after the scratch) and after 48 h of fixed cells stained with violet crystal. The bar size is 100 µm.

The activity of the EGF growth factors was further evaluated by scratch wound assays using the fibroblast NDFH cell line. This assay indicates the capacity of proliferation and migration of fibroblasts. Artificial wounds were created by scratching confluent layers of NDFH cells, following by photographing the wound gap of the live cultures and addition of 50 ng of each recombinant EGF growth factor. The cultures were incubated after 48 h, stained with crystal violet and photographed again. The percentage of scratch closure was determined by the ratio between the area of the gap at the beginning of the experiment and the area of the gap after 48 h (Fig. 7). The rate of artificial wound closure represents the combined induction of proliferation and migration. All EGF domains strongly induced wound closure when compared with the control. hEGF showed the highest rate of closure, followed by hEPR, hEPGN, hAREG, hBTC, hTGFα and, hHBEGF showing the lowest closure percentage. In summary, the activity analyses show that the recombinant EGF growth factors are highly active, being able to induce cell proliferation and migration.

## Discussion

The members of the EGF family of growth factors share the same structural domain, EGF, and are able to interact with cellular receptors and activate pathways leading to proliferation of different cells and tissues. The presence of three disulfide bonds in the EGF domain, with alternate combination for the first two pairs, renders a protein structure that is difficult to reconstitute in a functional conformation in prokaryotic organisms. In this work, we have developed genetic constructs and described detailed procedures to obtain seven human growth factors of the EGF family in a simple and easy fashion, using a single protocol and the same experimental set up and reagents. The genetic constructs containing the growth factors fused to Trx in pET32a proved to be very efficient for soluble expression in the *E. coli* Shuffle strain. The subsequent steps of affinity purification, thrombin digestion and recovery of the cleaved growth factors have worked very efficiently for all EGF growth factors tested. Our data show that utilization of Trx as fusion partner is very practical not requiring time-consuming methods for refolding in vitro of *E. coli* expressed insoluble proteins neither the use of eukaryotic cells as expression systems.

**Figure 8:**
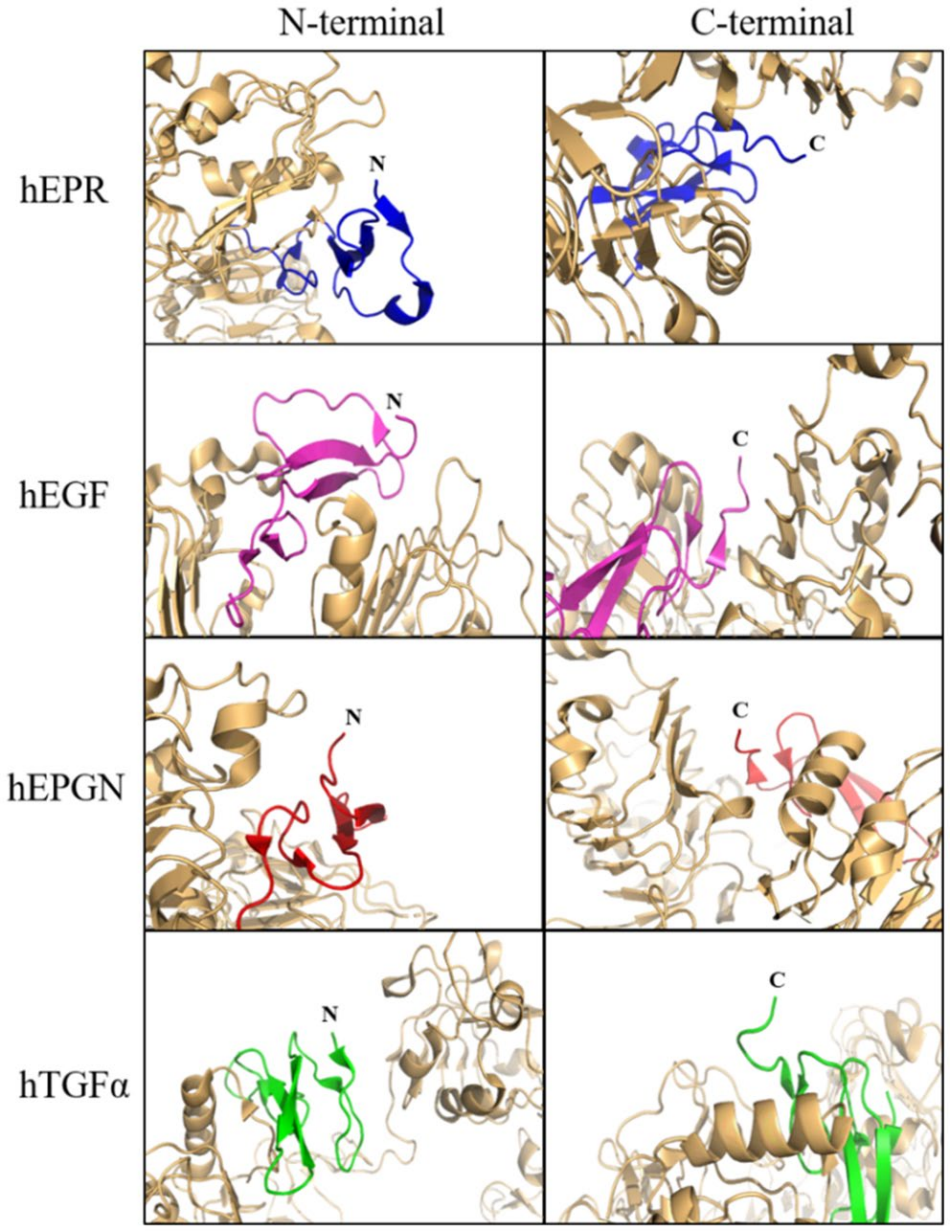
Analysis of the position of the N- and C-terminal ends of EGF growth factors bound to EGF receptors according to the crystal structures of the complexes available at PDB. The EGF receptors are shown in light orange. EGFR and EPR (blue) from PDB: 5WB7. EGFR and EGF (pink) from PDB: 1NQL.EPGN (red) from PDB: 5WB8. EGFR and TGFα (green) from PDB: 1MOX. The N- and C-terminal of the ligands are indicated. The images shown that the N- and C-terminals of the ligands are exposed to the solvent and not involved in the interaction with the receptor.

Concerning the N-terminal extension of the linker encoded by the vector pET32a that remains attached to the growth factor after digestion with thrombin, we have examined the binding mode of hEGF, hEPGN, hEPR and hTGFα to the receptor using the structures of their respective complexes with the extracellular domain of the EGF receptor deposited at PDB^39–41^. In all cases, the N- and C-terminal ends of the ligands are located outside the binding cavity, far from the binding interface, in positions which N- and C-terminal extensions are unlikely to interfere with binding (Fig. 8).

A major challenge for *E. coli* expression of small disulfide-bond-rich proteins is the correct pairing of the cysteine residues for disulfide bond formation so that the protein can assume a native folding. Previous studies have shown that Trx and SUMO were successfully used as fusion partners for *E. coli* expression of hEPR^22,26^ and hHBEGF^25^, respectively. However, it is important to point out that for expression of hEPR, an extension towards the N-terminus of pro-EPR needs to be included in the fusion protein for stability. The protein amenable to crystallization includes 25 residues of the propeptide^26^. Expression of the EGF domain of hEPR without N-terminal extension resulted in unstable protein (data not shown). Therefore, in order to improve the success rate aiming both soluble expression and stable protein after cleavage of Trx, the N- and C-terminal limits for cloning the EGF growth factors was based on the 3D structures deposited at PDB (Table 1).

**Table 1:** Sequences of the growth factors used in this study.

Growth factor	Uniprot code	Amino acid range	PDB code
hAREG	P15514	101–198 (188–198 part of the propeptide)	2RNL
hBTC	P35070	32–111	1IP0
hEGF	P01133-1	971–1023	1JL9
hEPGN	Q6UW88-1	49–109	5WB8
hEPR	O14944	38–108 (38–62 part of the propeptide)	5E8D
hHB-EGF	Q99075	63–148	1XDT
hTGFα	P01135-1	39–89	2TGF

Conformational and stability analyses were performed by indirect and direct methods to evaluate the folding state of the recombinant growth factors. Limited proteolysis and dynamic light scattering, which were performed in parallel with non-reduced and reduced samples, revealed striking differences between the two states. In the non-reduced state, all EGF growth factors showed much higher resistance to proteolysis, as well single peaks for radius size distribution at 20 °C, which are indications that the disulfide bonds are correctly formed. Interestingly, hAREG showed a divergent behavior under reducing conditions. DLS analyses indicated that hAREG does not aggregate after disulfide bond reduction even after long incubation times while all others showed aggregation under reducing conditions, although HBEGF and TGFα required longer times. Assays with increasing temperatures monitored both by nanoDSF and CD, did not detect clear transition states, which is consistent with the stable nature of the EGF domain sustained by the three disulfide bonds. The CD spectra of hEGF and hTGFα were similar to the CD spectra described in the literature^42^ for these factors and are consistent with spectra of proteins with none or low alfa helical content. hEGF, hEPGN, hEPR and hTGFα also showed strong resistance to aggregation under high temperatures while hBTC, hAREG and hHBEGF showed intermediate resistance to aggregation under heating. The conformational and stability analyses were complemented by mass spectrometry, which revealed the three correct disulfide bonds for AREG and correct bonds for the C1-C2 cysteine pairs of BTC, and C5-C6 for EPGN and EPR (Supplementary Figures S4–S7). The mass spectrometry analysis seems to be limited mostly by the low efficiency of the proteolytic digestion of the EGF domain, which does not generate di-peptides carrying disulfide bonds that are suitable for MS/MS.

The biological activity of the recombinant EGF growth factors was demonstrated by cell proliferation and migration assays. All of them induced cell proliferation at concentrations as low as 5 ng/mL. The cell proliferation effect obtained in our work for hEGF, hAREG, hBTC, hHBEGF and hTGFα is similar to the results published in previous studies for HeLa cells^43,44^, and in the case of hEGF also for other cell lines^45^. Considering the concentrations between 5 and 50 ng mL required for induction of proliferation, our findings for hEGF are similar to the ones described for MIO-M1 cell line^46^ and, for hEPGN and hTGFα described for myeloma cells stably expressing ErbB-1^45^. In addition to proliferation, the recombinant EGF growth factors can induce cell migration, as shown by the artificial scratch wound healing assays using NDFH cells. This finding is consistent with previous reports showing that hAREG, hHBEGF and hEGF are capable of inducing closure of scratches on other types of cell lines^46–49^. In general, the activity assays show that the recombinant EGF growth factors are highly active. This, together with the detailed information provided about the genetic constructs, purification procedures and biophysical analyses for quality control of folding state of the recombinant growth factors represent handy tools for quick and reliable in-house production of the seven members of the EGF growth factor family.

## Methods

### Plasmid constructs

The EGF domain of hAREG, hBTC, hEGF, hEPGN, hHBEGF and hTGFα were identified using Uniprot annotations and PDB structures, as shown in Table 1. Synthetic genes for these six factors were obtained from *GenScript* (Piscataway, NJ, USA) optimized for expression in *Escherichia coli* and cloned into the *Nco*I and *Xho*I restriction sites of plasmid pET32a^50^. The hEPR synthetic gene was designed as previously described by Kado and co-workers^26^ and acquired from GenCust (Boynes, France).

### Protein expression

All pET32a-derived plasmids were transformed into the *E. coli Shuffle* strain^30^ using heat shock^51^. Transformed cells were grown on LB-agar^52^ plates containing ampicillin (100 µg/mL) and incubated overnight at 37 °C. A single colony from each clone was used to inoculate 10 mL of LB containing ampicillin (100 µg/mL) and incubated overnight at 37 °C. 5 mL of this culture were used to inoculate 500 mL of LB containing ampicillin (100 µg/mL). When the O.D. at 600_ nm_ reached 0.6, the incubation temperature was reduced to 16 °C and expression induced by adding 0.5 mM IPTG. The incubation was continued for further 24 h and the cells harvested by centrifugation (6000 × g for 10 min) and stored at -80 °C.

### Protein purification

For purification, the cell pellet of 500-mL cultures was suspended in 10 mL of Buffer A (50 mM Tris.HCl pH 8.0; 300 mM NaCl; 20 mM imidazole) and the cells lysed using an M-110L microfluidizer (Microfluidics, Newton, MA, USA). After ten cycles of 18,000 psi, the extracts were clarified by centrifugation at 20,000 × g for 30 min. The supernatant was transferred to a 50 ml super-loop and assembled onto an ÄKTA Pure M25 chromatography system (Cytiva, Marlborough, USA). The recombinant proteins were initially purified by affinity chromatography using a 5 mL HisTrap column (Cytiva 17524802) pre-equilibrated in buffer A. The flow rate for all steps was 5 mL/min. After loading the sample, the column was washed with 3 column volumes (CV) of buffer A, followed by a gradient from 0 to 10% of Buffer B (50 mM Tris.HCl pH 8.0; 300 mM NaCl; 500 mM imidazol) in 2 CV and, the protein eluted using a 10 to 100% Buffer B gradient in 4 CV. Fractions of 1 mL were collected and the peak fractions analyzed by SDS-PAGE.

For digestion with thrombin, a 5 mL volume of peak samples was diluted to a final volume of 15 mL using dilution buffer (25 mM Tris.HCl pH 8.0; 150 mM NaCl). The digestions were performed using 1 U of thrombin (Cytiva 27084601) for 10 mg of recombinant fusion protein (Trx-EGF factor) at 25 °C for 24 h. Once the digestion was confirmed by SDS-PAGE, the products of digestion were loaded onto a 50 mL super-loop and the his-tagged Trx was captured on a 5 mL HisTrap column (Cytiva 17524802) pre-equilibrated in buffer A using an ÄKTA Pure M25 system (Cytiva, Marlborough, MA, USA). After sample injection, the column was washed using 3 CV of Buffer A. The flowthrough containing the EGF growth factor was collected and concentrated using Amicon Ultra-0.5 mL 3 K concentrators up to a final volume of 500 µL. Subsequently, the concentrated 500-µL samples were fractionated by size exclusion chromatography. For this purpose, a Superdex 75 10/300 GL (Cytiva 29148721) was equilibrated with size exclusion buffer (25 mM Tris.HCl pH 8.0; 150 mM NaCl) using an ÄKTA Pure M25 system (Cytiva, Marlborough, MA, USA) at flow rate of 0.8 mL/min. Fractions of 0.5 mL were collected and samples from the chromatographic peak fractions were analyzed by SDS-PAGE as described below.

### SDS-PAGE analysis of proteins

Cells extracts, chromatography fractions, purified proteins and proteolysis reactions were analyzed by electrophoresis using the Laemmli buffer system^53^. Poly-acrylamide concentrations in the stacking and separating gels were 6.5% and 15%, respectively. 0.75 mm-thick gels were assembled on Mini-PROTEAN Tetra Cells (Bio-Rad Laboratories, Hercules, USA). Protein samples were mixed with Laemmli sample buffer, heated to 95 °C for 5 min and applied onto the gel wells. Electrophoresis was performed at 100 V. Gels were stained using Coomassie Brilliant Blue R-250 and destained using an methanol/acetic acid/water (30/10/60) solution^54^.

### Limited proteolysis assay

Limited proteolysis was performed with the recombinant growth factors under reducing and non-reducing conditions. For reduction of the disulfide bonds, the growth factors were treated with 10 mM DTT (Merck 3483123). The samples were incubated with 0.1% (w/w) trypsin (Sigma-Aldrich 59427C) for 30 and 60 min at 37 °C. Controls without trypsin were incubated for 60 min. The products of digestions and control reactions were analyzed by SDS-PAGE as described above.

### Dynamic Light Scattering and effect of temperature monitored by nano-differential scanning fluorimetry (nanoDSF)

The purified recombinant growth factors were analyzed on a nanoDSF Prometheus Panta device (NanoTemper Technologies, Munich, Germany) for DLS and thermal effect analyses under reducing and non-reducing conditions using standard capillaries. DLS measurements were performed at 20 °C using the high sensitivity setup (10 acquisitions of 5 s each) and two independent capillaries. For analysis of the thermal effect, the samples without reducing agents were subjected to a linear thermal ramp from 20 to 95 °C with parallel measurement of DLS.

### Circular dichroism

For circular dichroism analyses, the sample buffer was changed by ultrafiltration to 4.2 mM Tris–HCl pH 8.0 and 25 mM NaCl using Amicon Ultra-0.5 mL 3 K concentrators. CD spectra were acquired using recombinant growth factors at 0.2 mg/mL in 1 mm path cuvette on a Jasco J-815 circular dichroism spectrometer (JASCO, Tokyo, Japan). The scanning speed was 50 nm/min. Each spectrum was accumulated at least five times. Spectra were acquired from 195 to 260 nm at 20 °C and after raising the temperature from 20 to 70 °C, 75° or 85 °C, depending on the voltage limit. A new set of spectra was acquired after cooling to 20 °C. The raw data were converted using the mean residue molar ellipticity formula and autozero was set by using the value at 250 nm.

### Cell proliferation assay

HeLa cells (Carlos Chagas Institute cell bank) were cultivated in DMEM (ThermoFisher 41965062) supplemented with 10% BFS until confluence using a Forma Series II Water Jacketed CO_2_ incubator (Thermo Fischer Scientific, Waltham, MA, USA). Cells were cultivated at 37 °C, with humidity and 5% CO_2_. Subsequently, 10^5^ cells per well were transferred to a 24 well plate and incubated for additional 24 h, when the medium was replaced by DMEM without BFS, and the cultures were treated with 50 ng/mL of recombinant EGF growth factors in triplicates in parallel with untreated controls also in triplicates. After 48 h, the wells were washed with PBS and treated with 200 µL of PBS containing trypsin 1% (w/v) (Merck T8003). After 5 min of trypsin action at 37 °C, the released cells were collected by centrifugation at 500 × g for 5 min and suspended in 280 µL of PBS and 20 µL of *CountBright™ Absolute Counting Beads* (ThermoFisher C36950). The cell number was determined on a BD FACS Canto II (BD Life Sciences, New Jersey, USA) and the results analyzed by FlowJo™ v10.8 (BD Life Sciences, Franklin Lakes, NJ, USA). At least 1000 beads were acquired and a maximum of 3000 beads was set as upper limit. Quantification was performed as described by the *CountBright™ Absolute Counting Beads* manual (Invitrogen MAN0018850).

For dose–response assays, 5 × 10^4^ HeLa cells (Carlos Chagas Institute cell bank) per well from a confluent culture were cultivated in 96-well plates for 24 h using a Forma Series II Water Jacketed CO_2_ incubator (Thermo Fischer Scientific, Waltham, MA, USA). The cells were cultivated at 37 °C, with humidity and 5% CO_2_. Subsequently, they were treated with five increasing concentrations of recombinant growth factors in triplicates: 0.005 ng/mL, 0.05 ng/mL, 0.5 ng/mL, 5 ng/mL and 50 ng/mL. After 48 h of incubation, the wells were washed with PBS, treated with 50 µL of MTT (Invitrogen M6494) at 5 mg/mL and 50 µL of serum free medium and incubated at 37 °C for 3 h. After this time, 150 µL of the solvent solution (4 mM HCl, 0.1% NP40 in isopropanol) was added and the incubation continued for another hour. Absorbance was read at 570 nm using a Synergy H1 Hybrid Reader (BioTek Instruments, Winooski, VT, USA). Dose–response curves were adjusted using nonlinear fit of the data.

### Scratch closure assay

NDFH cells (Lonza CC-2509) were cultivated in DMEM (ThermoFisher 41965062) supplemented with 10% BFS until confluence using a Forma Series II Water Jacketed CO_2_ incubator (Thermo Fischer Scientific, Waltham, MA, USA). Cells were cultivated at 37 °C, with humidity and 5% CO_2_. Subsequently, the medium was changed to DMEM without BFS, and the incubation continued for another 24 h. At this time, a scratch was made, 50 ng of each recombinant factor was added to cultures in triplicates and images of the unstained cells were acquired. After 48 h of incubation, the cells were stained using crystal violet (Synth C.I. 42555) according to the supplier’s instructions. Briefly, the cells were fixed using ice-cold 100% methanol for 10 min. Afterwards, 0.1 mL of a 0.5% crystal violet solution in 25% methanol was added to each well, incubated for 10 min and washed with PBS. Images of the cells were acquired at time 0 and 48 h after the treatment with the growth factors on a Nikon Eclipse TE300 Inverted Microscope (Nikon, Tokyo, Japan) using a 10X objective and the QCapture Pro 6.0 software (Teledyne QImaging, Surrey, Canada). The rate of artificial wound healing was calculated using ImageJ^55^. The scratches were aligned with the base of the picture and a 900 × 600-pixel rectangle was cropped around the scratch. The area at time 0 was calculated using the polygon selection tool and the area at 48 h was calculated using the area between the edges of the scratch area converted to 8-bit images and sharpened. The find edges tool was used and the image was converted to binary. The particles were then analyzed using sizes between 10 and 100,000 with bare outlines, including holes to calculate the areas.

### Statistical analysis

Graphs and statistical analyses were performed using the GraphPad Prism 5 software (Graph Pad, San Diego, USA). The data from assays in triplicate for each growth factor were compared to the control data using One-Way analysis of variance and Dunnett posttest, where p < 0.05 was considered statistically significant.

### Analysis of protein 3D structures

The structures of the EGF growth factors and of the complexes of EGF growth factors and EGF receptors were visualized using PyMOL Molecular Graphics System version 4.5.0, Schrödinger, LLC.

## Supplementary Information

Supplementary Information 1.

Supplementary Information 2.

## Data Availability

Original datasets and reagents generated during this study are available from the corresponding author on reasonable request.
