# Supplementary material for: A toolkit for recombinant production of seven human EGF family growth factors in active conformation
Source: Sci Rep. 2022 Mar 23;12:5034. doi: 10.1038/s41598-022-09060-9 (PMC8943033; doi:10.1038/s41598-022-09060-9)
Supplement: Supplementary file 2 — Supplementary Information 2. [file 41598_2022_9060_MOESM2_ESM.pdf]

Supplementary Information to the paper

A Toolkit for Recombinant Production of all Seven Human EGF Family Growth Factors in Active Conformation

Arthur Schveitzer Ferreira [1,2]; Amanda Lopacinski [1,2], Michel Batista [3], Priscila Mazzocchi Hiraiwa, Beatriz Gomes Guimarães [1], Nilson Ivo Tonin Zanchin [1,2, #]

[1]Laboratory of Structural Biology and Protein Engineering, Carlos Chagas Institute, FIOCRUZ Paraná – Curitiba-PR, Brazil

[2]Cellular and Molecular Biology Graduate Program, Federal University of Paraná – Curitiba-PR, Brazil.

[3]Mass Spectrometry Facility RPT02H, Carlos Chagas Institute, FIOCRUZ Paraná – Curitiba-PR, Brazil.

# Corresponding author. Email address: nilson.zanchin@fiocruz.br

Supplementary information content:

1. Complete images of the polyacrylamide gel electrophoresis analyses performed during the purification of the EGF growth factors.

2. Complete images of the polyacrylamide gel electrophoresis analyses of the limited proteolysis assays.

3. NanoDSF plots showing the effect of temperature increase on the seven EGF factors.

4. Mass spectrometry analysis of disulfide bonds

1. Complete images of the polyacrylamide gel electrophoresis analyses performed during the purification of the EGF growth factors.

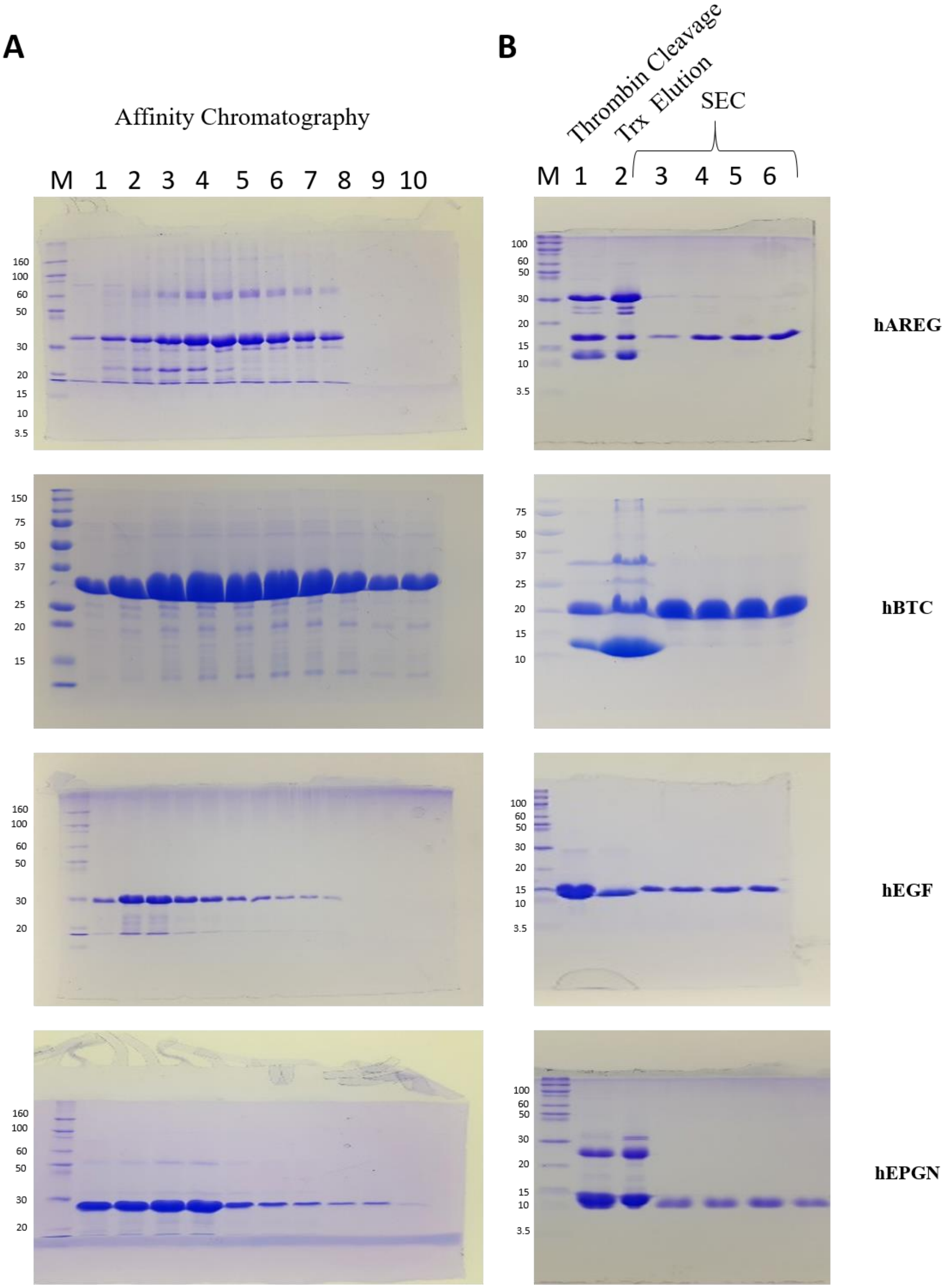

Continued on the next page.

Continued

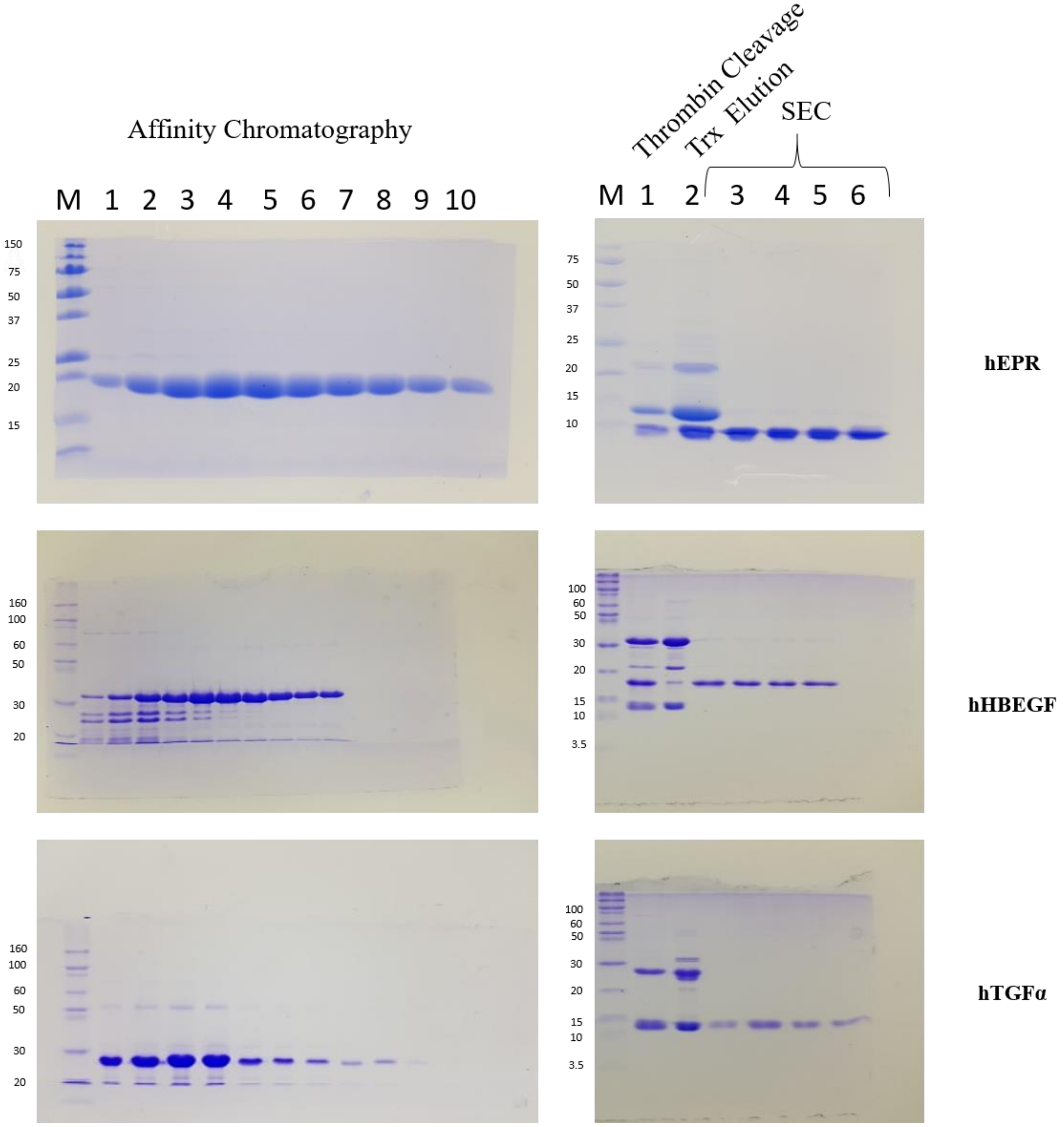

Figure S1. Complete images of the analyses performed during the purification of the EGF growth factors **(A)** Original images of the full-length Coomassie stained SDS-polyacrylamide gels showing the peak fractions of the initial affinity chromatography. **(B)** Original Images of the full-length Coomassie stained SDS-polyacrylamide gels showing the result of thrombin protease digestion (thrombin), the separation of Trx-His6 by capture on an IMAC column (Trx-His6) and the size exclusion chromatography. 1, products of the thrombin protease digestion; 2, elution fraction from the purification of Trx-his6 after thrombin cleavage; 3-6, peak fractions after the size exclusion chromatography (SEC) of the growth factors. M, molecular mass markers. Masses in kDa are indicated on the left.

2. Complete images of the polyacrylamide gel electrophoresis analyses of the limited proteolysis assays.

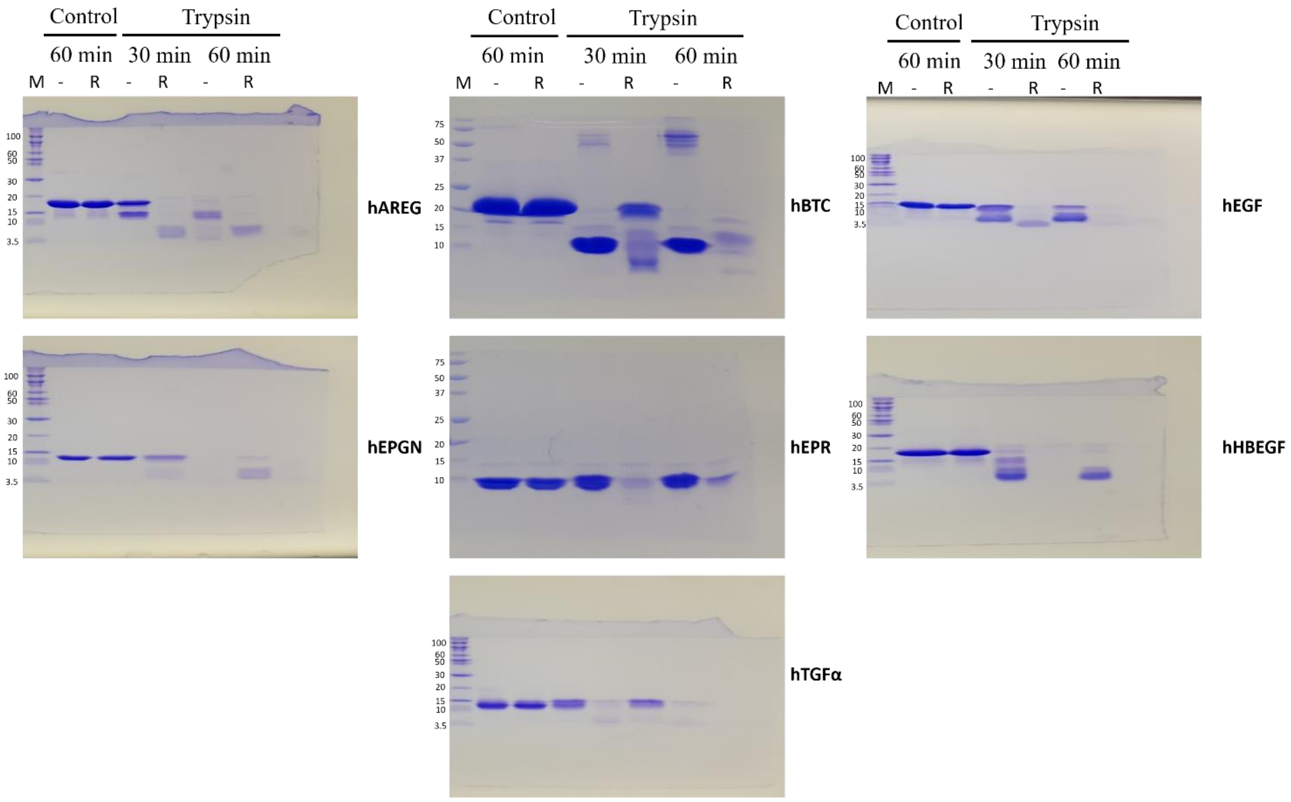

Figure S2. Complete imagens of the Coomassie stained SDS-polyacrylamide gels used for the analysis of the proteolysis products and control reactions of the EGF growth factors as identified on the right side of each panel. Stability analysis by limited proteolysis using trypsin. The proteolytic assays were performed with samples both untreated (-) and treated with the reducing agent DTT and incubated for 30 and 60 min. Control non-reduced and reduced samples without trypsin treatment were incubated under the same conditions. M, molecular mass markers. Masses in kDa are indicated on the left. -, non-reducing condition. R, reducing condition.

3. NanoDSF plots showing the effect of temperature increase on the seven EGF factors

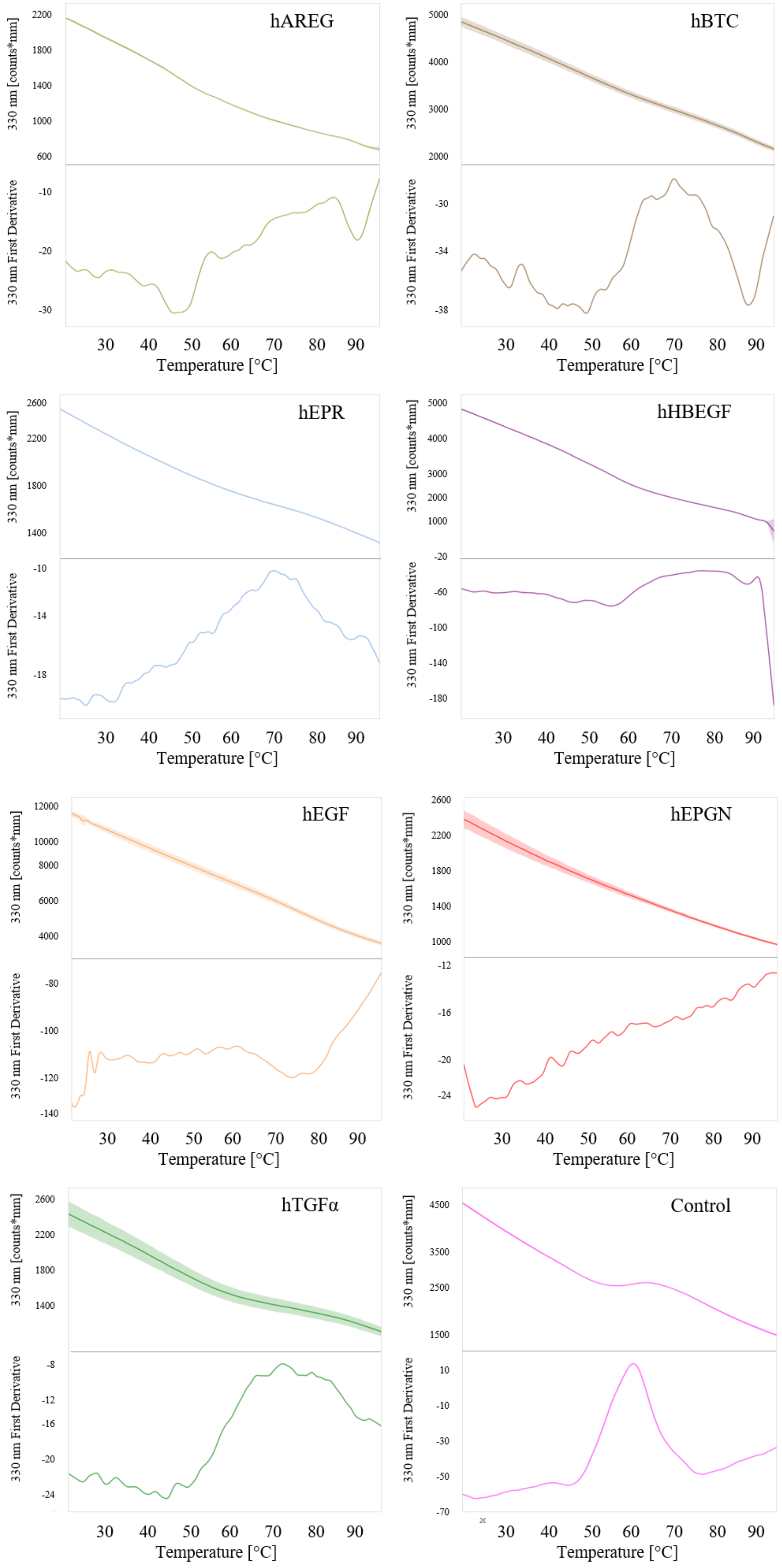

Figure S3. Effect of temperature increase on the seven EGF factors monitored by nanoDSF. Top and bottom panels show, respectively, the fluorescence at 330 nm as a function of temperature and its first derivative. In contrast to the control sample (bottom right), a transition temperature could not be determined for the growth factors.

4. Mass spectrometry analysis of disulfide bonds

The seven recombinant growth factors were digested without reduction of cysteines using different proteases selected according to the expected cleavage sites present in their primary sequence and the peptides were analyzed by LC-MS/MS methods with the combinations described below:

Factor	Proteases	LC-MS/MS
AREG	Lys-C plus Glu-C	nano LC EThcD, ESI LC HCD
BTC	Lys-C plus Glu-C	nano LC EThcD, ESI LC HCD, nano LC HCD
EGF	Lys-C plus Glu-C	nano LC EThcD, ESI LC HCD, nano LC HCD
EPGN	Lys-C plus trypsin, plus pepsin	nano LC EThcD
EPR	Pepsin plus trypsin	nano LC EThcD
HBEGF	Lys-C plus pepsin; Lys-C plus Glu-C	nano LC EThcD, ESI LC HCD, nano LC HCD
TGFa	Lys-C plus pepsin	nano LC EThcD

For peptide preparations, 5 µg of unreduced recombinant EGF domain factors were incubated in 6 M urea, 5 mM NEM for 15 min at RT, digested for 4 h at 37 °C with 0.1 µg Lys-C (except EPR), diluted with protease compatible buffer and incubated with another protease. The protease/EGF domain factor ratios ranged from 1:20 to 1:50. The second or third proteolytic reactions were incubated at 37 °C overnight and inactivated by acidification or by heating. Proteolysis products were desalted using C18 stage tips and the eluates from the stage tips concentrated by speed vac. Hundred nanograms of digested peptides were submitted to LC-MS/MS using an Ultimate 3000 RSLCnano coupled to an Orbitrap Fusion Lumos (Thermo Fischer Scientific, Waltham, MA, USA). The liquid chromatography analyses were performed using a flow of 100 µL/min (ESI LC HCD) or 250 nL/min (nano LC HCD) and gradient of 5 to 40% MeCN, 0.1% formic acid for 90 min. Data acquisition was performed in the DDA mode with MS1 and MS2 (post HCD fragmentation) spectra acquired with the orbitrap set to 120k resolution with automatic gain control of $4x10^5$ and maximum injection time of 200 ms or 240 ms, respectively. In the nano LC EThcD analyses, gradients from 5 to 40% MeCN, 0.1% formic acid were carried out for 30 min. MS1 spectra were acquired in the DDA mode with the orbitrap set to 120k resolution with automatic gain control standard and maximum injection time of 50 ms. Fragmentation was performed using EThcD MS2 with the orbitrap set to 30k resolution using automatic gain control standard, and maximum injection time of 54 ms.

Raw data were analyzed using BioPharma Finder 3.0 and the disulfide bond sites identification was required to fit the follow criteria: up to 3 disulfide bonds per ion; MS2 identifications, precursor mass error ≤ 5 ppm and, confidence score > 0. With these criteria, we have directly identified di-

peptides linked by a single disulfide bond for AREG, BTC, EPGN, and EPR (Figures S4 to S7). Unfortunately, it was not possible to map disulfide bonds for EGF, HBEGF and TGFα. EGF and HBEGF analyses mapped bonds (C38-C52 for EGF, and C78-C91 for HBEGF) that did not reach the requirements of the criteria described above. According to the LC-MS/MS chromatogram, EGF seems to be more resistant to digestion than the other factors. HBEGF and TGFα were digested with pepsin combined with Lys-C or Trypsin. However, since pepsin can use a relatively high number of amino acid residues as cleavage sites and at different rates, the digestion efficiency did not correlate with peptide identification, hampering mapping of disulfide bonds. The precise mapping of all predicted disulfide bonds present in the seven growth factors here evaluated is an overwhelming challenge. A possible way to get through this, may require the digestion of milligram amounts of each factor, followed by multiple cycles of digestion and purification of the peptides to obtain di-peptides carrying a single disulfide bond, that can be unambiguous identified by LC-MS/MS.

AREG

Amino acid sequence:
GSGMKETAAAKFERQHMDSPDLGTDDDDKAMA**SVRVEQVVKPPQNKTESENTSDKPKRKKKGGKNGKNRRNRK**
KKNPCNAEFQNFCIHGECKYIEHLEAVTCKCQQEYFGERCGEKSMKTHSMIDSSLSKL

Predicted disulfide bonds: C78-C91, C86-C102, C104-C113

Sites identified:

Site	Modification	Delta (ppm)	Confidence Score	ID Type	MS Area	method	Identification
1:C104,C113	1ss	-0.39	100	MS2	6.79E+07	nano LC EThcD	C104-C113 MS2 confirmatory spectrum
1:C102/1:C78,C86,C91	2ss	0.18	91.9	MS2	1.54E+07	nano LC EThcD	C78-C91 by inference
1:C102/1:C86	1ss	-0.08	100	MS2	2.67E+04	ESI LC HCD	C86-C102 MS2 confirmatory spectrum

C104-C113 and C86-C102 were identified directly by MS2 confirmatory spectra. C78-C91 was identified by inference since it is part of a di-peptide containing a directly identified bond.

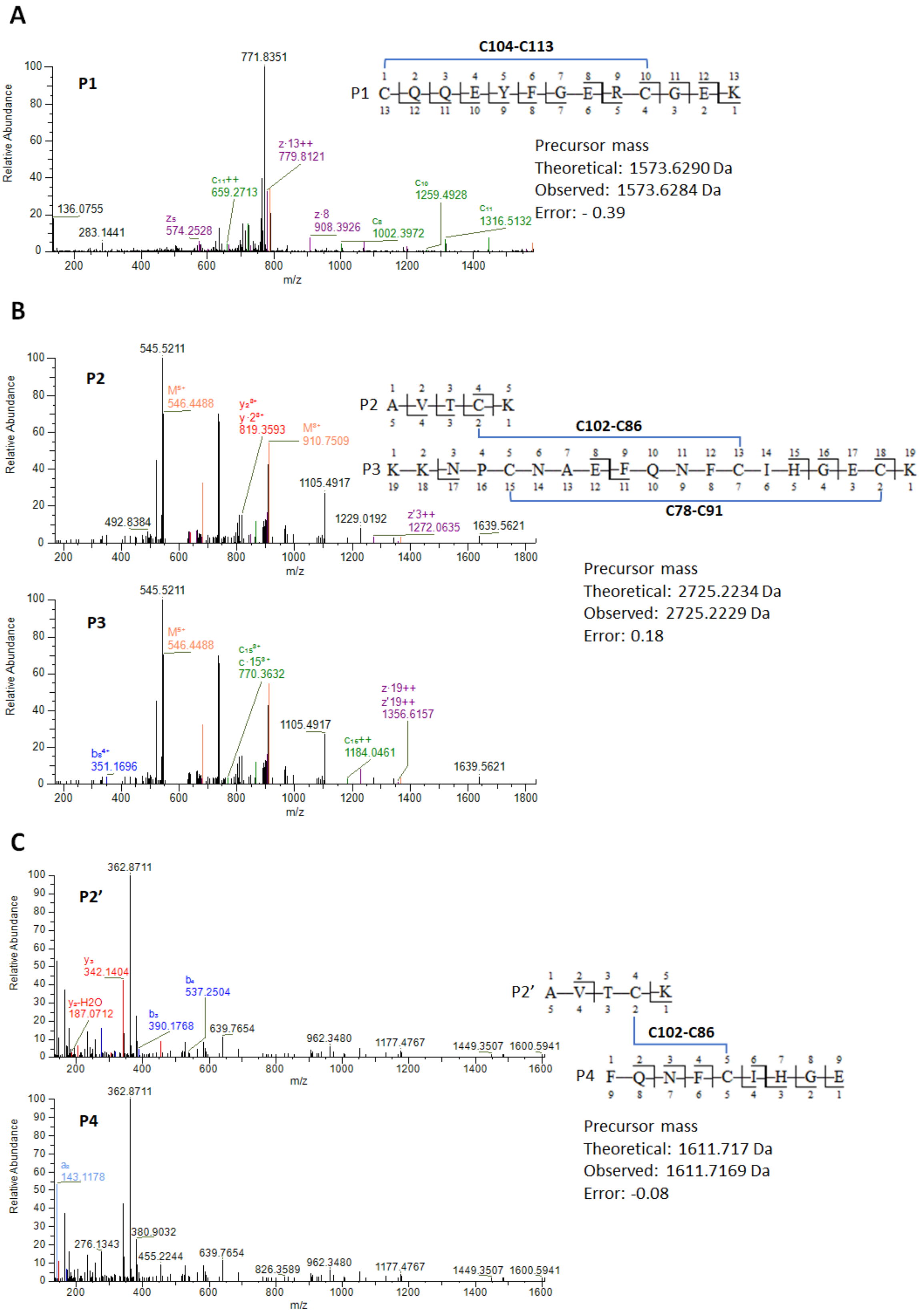

Figure S4. Assignment of the MS/MS spectra of the disulfide bond-containing peptides of the recombinant AREG EGF domain. The three disulfide bonds C104-C113 (A), C102-C86/C78-C91 (B) and C102-C86 (C) were identified by MS/MS. Peptides P2 and P3 were identified in the same spectrum (B), however they were annotated separately. The same is valid for P2’ and P4 (C).

BTC

Amino acid sequence:

GSGMKETAAAKFERQHMDSPDLGTDDDDKAMA**DGNSTRSPETNGLLCGDPEENCAATTTQSKRKGHFSRCPKQ YKHYCIKGRCRFVVAEQTPSCVCDEGYIGARCERVDLFY**KL

Predicted disulfide bonds: C70-C83, C78-C94, C96-C105

Site identified:

Site	Modification	Delta (ppm)	Confidence Score	ID Type	MS Area	method	Identification
1:C70/1:C83	1ss	0.12	98.6	MS2	4.25E+08	nano LC EThcD	C70-C83 MS2 confirmatory spectrum

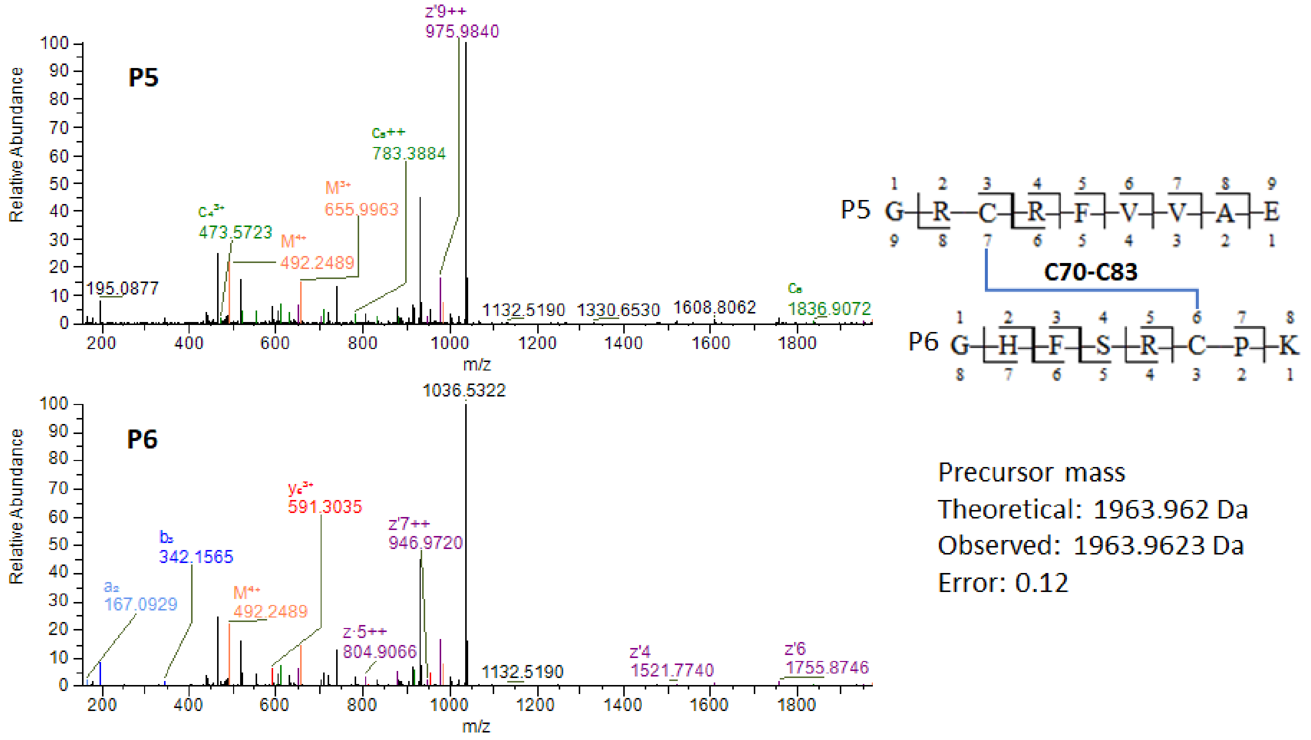

Figure S5. Assignment of the MS/MS spectrum of the disulfide bond-containing peptide of recombinant BTC. The disulfide bond C70-C83 was identified by MS/MS. Peptides P5 and P6 were identified in the same spectrum, however they were annotated separately.

EPGN

Sequence:

GSGMKETAAAKFERQHMDSPDLGTDDDDKAMAEGPIALKFSHLCLEDHNSYCINGACAFHHELEKAICRCFTG
YTGERCEHLTLTSYAVDSYE

Predicted disulfide bonds: C44-C57, C52-C68, C70-C79.

Site identified:

Site	Modification	Delta (ppm)	Confidence Score	ID Type	MS Area	Method	Identification
1:C70,C79	1ss	-0.78	100	MS2	9.94E+07	nano LC EThcD	C70-C79 MS2 confirmatory spectrum

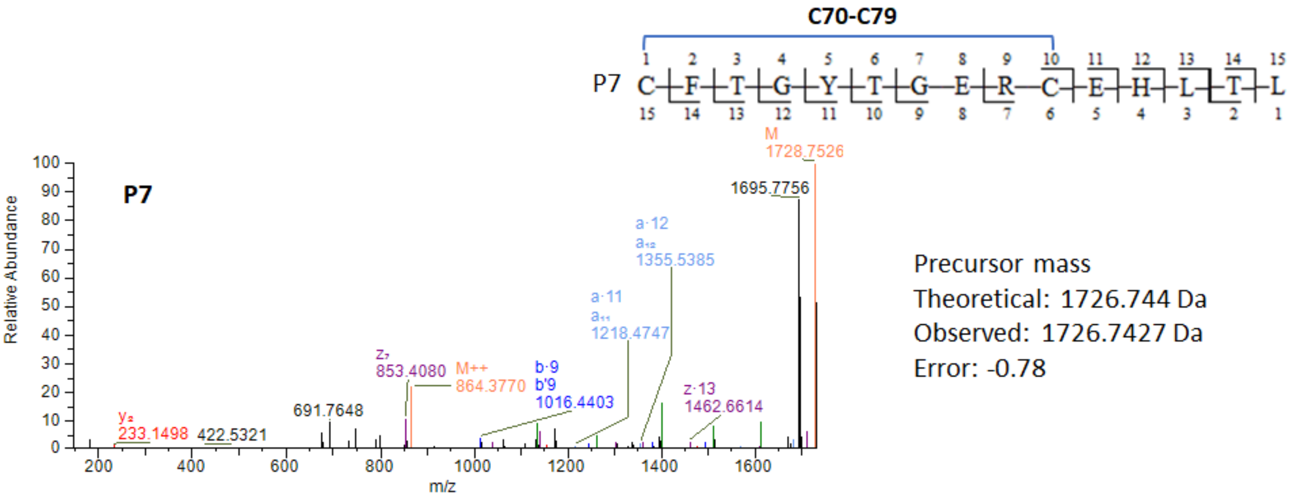

Figure S6. Assignment of the MS/MS spectrum of the disulfide bond-containing peptide of recombinant EPNG. The disulfide bond C70-C79 was identified by MS/MS.

EPR

Amino acid sequence:

GSGMKETAAAKFERQHMDSPDLSCIPGESSDNCTALVQTEDNPRVAQVSITKCSSDMNGYCLHGQCIYLVDMS QNYCRCEVGYTGVRCEHFFL

Predicted disulfide bonds: C53-C66, C61-C77, C79-C88

Site identified:

Site	Modification	Delta (ppm)	Confidence Score	ID Type	MS Area	Method	Identification
1:C88/1:C79	1ss	-0.96	71	MS2	1.55E+06	nano LC EThcD	C79-C88 MS2 confirmatory spectrum

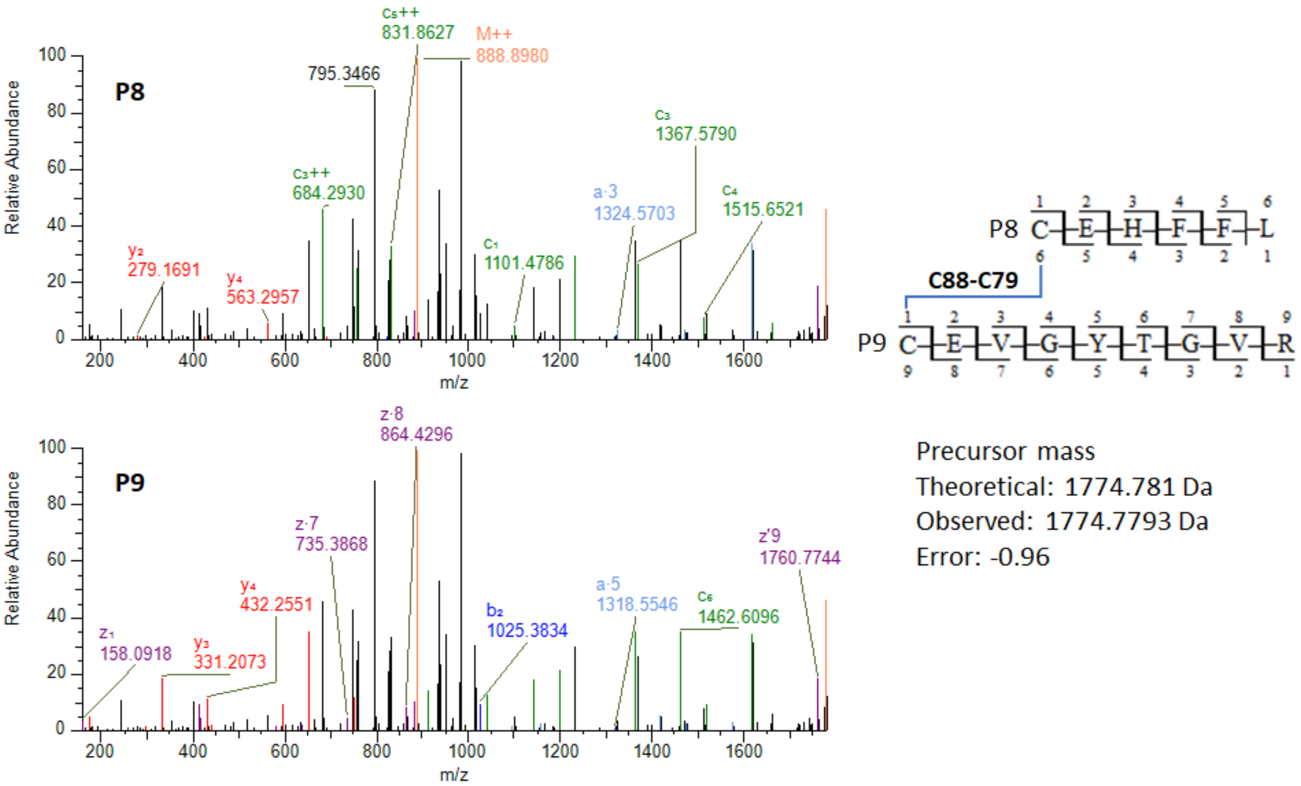

Figure S7. Assignment of the MS/MS spectrum of the disulfide bond-containing peptide of recombinant EPR. The disulfide bond C79-C88 was identified by MS/MS. Peptides P8 and P9 were identified in the same spectrum, however they were annotated separately.
